# FeXAI: Federated and Explainable AI for cyber threat detection in IoT-enabled smart transportation systems

**DOI:** 10.1038/s41598-026-46141-5

**Published:** 2026-04-09

**Authors:** Abirami Gurushanker, A. Jeffrey Rufus, C. Christopher Columbus, C. K. Aravind

**Affiliations:** 1 School of Computer Science and Engineering, Vellore Institute of Technology, Chennai, Tamil Nadu India; 2 School of Electrical Engineering, Vellore Institute of Technology, Chennai, Tamil Nadu India

**Keywords:** Explainable AI, Federated learning, Internet of Things, Internet of vehicles, Intrusion detection systems, Machine learning, Smart city, Vehicular Ad hoc Networks, Energy science and technology, Engineering, Mathematics and computing

## Abstract

The rapid development of smart cities, fueled by the growth of the Internet of Things (IoT) and interconnected systems, has greatly enhanced urban infrastructure, especially in transportation and energy management. However, this increased connectivity also raises the risk of cyberattacks, threatening service availability, financial stability, and public safety. This study introduces a resilient cybersecurity framework designed to detect and classify various cyber threats, including DoS, DDoS, Reconnaissance, Sybil, Replay, and Spoofing attacks, targeting critical transportation systems such as the Internet of Vehicles (IoV), electric vehicle (EV) charging networks, and Vehicular Ad hoc Networks (VANETs). By combining machine learning with Federated Learning (FL), the framework effectively tackles key challenges like high computational costs, dependence on centralized data, and scalability across different IoT systems. FL improves data privacy by keeping sensitive information on edge devices, reducing concerns over centralized data storage. Moreover, TreeSHAP, an interpretability technique, is utilized to provide transparency and deeper insights into attack detection. The proposed system achieves high F1 scores of 0.980, 0.982, and 0.99 on the CICIoV2024, CICEVSE2024, and VeReMi Extension datasets, respectively, demonstrating its effectiveness on multiple IoT security datasets relevant to smart city transportation and energy systems. while safeguarding user privacy.

## Introduction

As urban environments quickly evolve with technological advances, the idea of smart infrastructure is gaining widespread global adoption. This change depends on connected networks and a wide variety of Internet of Things (IoT) devices to improve and optimize key services like transportation and energy management^1^. These systems boost efficiency, sustainability, and overall service quality by using real-time data collection and advanced analytics. Better resource management, lower costs, and improved user experience are some of the main benefits of these innovations^2,3^. However, the greater connectivity in these infrastructures also brings significant security challenges, especially in critical sectors such as transportation and energy. Disruptions in these systems can have serious consequences, impacting economic stability, public safety, and the reliability of services^4^.

Critical infrastructures, including electric vehicle (EV) charging networks, the Internet of Vehicles (IoV), and Vehicular Ad hoc Networks (VANETs), are becoming increasingly reliant on IoT technology^5^. While these systems improve traffic management, facilitate seamless vehicle-to-vehicle communication, and optimize energy distribution, they also enlarge the attack surface for cyber threats^6^. Many IoT devices in these networks lack strong security measures, making them vulnerable to exploitation. Cyberattacks on these infrastructures can result in serious consequences, such as service disruptions, financial losses, privacy breaches, and threats to public safety^7^. Given these risks, protecting these systems has become a top priority for researchers, developers, and policymakers^8^. A robust Intrusion Detection System (IDS) is one of the most essential tools for safeguarding IoT-based infrastructure, playing a key role in cybersecurity^9^. These systems monitor network traffic in real time, detect anomalies, and identify potential security breaches, maintaining the integrity and security of interconnected networks and devices^10,11^. Considering the complexity and large scale of transportation and energy networks, IDS solutions designed for these critical sectors must be highly advanced, capable of detecting and countering sophisticated cyber threats before they cause significant harm^12^.

To address these challenges, existing research has mainly concentrated on deep learning-based methods for creating attack detection systems^13^. Deep learning models, especially neural networks, have shown outstanding performance in detecting patterns in large datasets and spotting complex cyber threats^14^. However, these models require large amounts of labeled training data and substantial computational power, making real-time implementation difficult. Furthermore, while deep learning is effective in anomaly detection and intrusion prevention, its ability to accurately classify attack types within smart city infrastructures remains limited^5,9^. Although some studies have explored machine learning-based approaches as alternatives, these methods have not yet delivered significant improvements in attack classification, which remains a major obstacle in cybersecurity^15^.

Beyond traditional machine learning methods, researchers have investigated Federated Learning (FL) and metaheuristic-based techniques to improve IDS performance^16^. FL provides a decentralized, privacy-preserving approach to machine learning, enabling IDS models to be trained across distributed networks without centralizing sensitive data^7^. Although promising, many FL implementations still depend on deep learning architectures^12^, which leads to challenges like data dependency, high computational costs, and limited adaptability within individual IoT applications such as transportation and energy systems^17^. Furthermore, much of the current research concentrates on specific subdomains, such as EV charging networks, IoV, or VANETs^18^. Consequently, solutions tend to be narrowly focused and lack the flexibility to accommodate dataset- or protocol-specific differences across IoT systems^19^. This fragmentation highlights a significant gap that motivates evaluating different datasets individually within a unified analytical framework.

To close this gap, there is an urgent need for a comprehensive and adaptable framework that effectively integrates and safeguards various IoT-based systems within transportation and energy networks. Such a framework must not only ensure real-time threat detection but also accurately classify cyberattack types across multiple domains^9^. Furthermore, it must include scalable, efficient, and low-latency solutions to keep up with the rapidly changing landscape of urban technology while maintaining strong security measures^16^.

The contributions of this research are as follows:

i. Experimenting with various IoT datasets to identify and optimize the most effective machine learning models for intrusion detection and attack classification in smart city environments.
ii. To develop a federated learning framework for privacy-preserving intrusion detection using separate datasets representative of smart city subsystems.
iii. To Integrate explainable artificial intelligence (XAI) techniques into the framework, enabling transparent and interpretable decision-making processes that give stakeholders actionable insights into detected threats.s.

## Related work

The Internet of Things (IoT) has become essential in various applications, playing a key role in modern systems, daily life, and business operations. IoT is especially important in managing the Internet of Vehicles (IoV), electric vehicle charging networks (EVCN), Electric Vehicle Supply Equipment (EVSE), and Vehicular Ad hoc Networks (VANETs). As IoT adoption grows, strong cybersecurity measures become increasingly critical. Cybersecurity involves technologies and practices that protect devices, platforms, and networks from threats like cyberattacks and hacking, ensuring overall system security. This section examines research in IoT, IoV, EVCN, and VANETs with an emphasis on cybersecurity. Machine Learning (ML) and Deep Learning (DL) are vital in modern cybersecurity by enhancing security measures. ML allows systems to analyze data and make predictions without explicit programming, while DL processes large volumes of raw data, enabling cybersecurity systems to learn independently.

### Cybersecurity in IoT networks

Damiano Torre et al.^20^ proposed a Federated Learning-based IDS (FL-IDS) to improve IoT network security. Using a one-dimensional convolutional neural network, it processes IoT data while integrating Differential Privacy, Diffie–Hellman Key Exchange, and Homomorphic Encryption to protect privacy. A 1D-CNN tested on seven IoT datasets, including TON IoT and IoT-23, achieves 97.31% accuracy, 95.59% precision, 92.43% recall, and 92.69% F1-score. Privacy techniques add 10% computational overhead while preserving security. Mahdi et al. propose a hybrid IDS combining LSTM with Naive Bayes for IoT security against DDoS and spoofing^4^. Their feature selection method combines correlation coefficient analysis and sequential selection to find relevant features. Tested on three datasets, the approach reaches 99.91% accuracy for IoT environments.

Merzouk et al. analyze the susceptibility of Deep Reinforcement Learning (DRL)-based Intrusion Detection Systems (IDS) to adversarial attacks^21^. Using NSL-KDD, UNSW-NB15, and CICIoV2024, they evaluate DRL algorithms, demonstrating that adversarial perturbations affect IDS accuracy, with vulnerability depending on configuration and methods. Ruoyu Li et al. introduced ADRIoT, an anomaly detection framework for IoT security^22^. It employs edge computing with LSTM autoencoders to identify threats without labeled attack data. The system includes a traffic capture, preprocessor, and anomaly detector on edge devices, utilizing multi-edge collaboration to optimize resources and protect resource-constrained devices.

### Cybersecurity in IoV

Neto et al. introduce CICIoV2024, a dataset for intrusion detection in vehicular networks, focusing on DoS and spoofing attacks on CAN bus^1^. Using a 2019 Ford vehicle, they collected attack data in multiple formats. ML models like Random Forest and Deep Neural Networks achieved 96% accuracy, although similar attacks were difficult to differentiate. Limitations include data from only one vehicle and dataset imbalance. Gül & Bakir propose a GA-enhanced IDS for IoV security^23^. Using ML models like Random Forest and AdaBoost, they achieved 99.64% accuracy, with GA tuning the model parameters. Lu et al. introduce IDM-DCFM, an intrusion detection model for the CAN bus utilizing deep convolutional autoencoders and factorization machines^24^. The model addresses limitations in capturing local features and high-order interactions within CAN messages. IDM-DCFM improves precision (2.2%), accuracy (1.5%), and F1-score (1.3%) compared to DNN-based models, though computational overhead impacts performance. Altalbe presents FFS-IDS, an ML-based IDS for in-vehicle networks^25^. Using stacking ensemble learning with decision trees as base classifiers and Random Forest as the meta-learner, it achieves 99% accuracy for DoS, Gear Spoofing, and RPM Spoofing, and 97.5% for Fuzzy attacks, outperforming traditional models.

Y. Yigit et al. propose an AI-driven digital twin framework for security and efficiency in 6G Internet of Vehicles (IoV)^26^. It uses stacked sparse autoencoders (ssAE) for feature reduction and online learning for real-time attack detection. The system has three layers: Data Layer for collecting real-time vehicle data; Cyber Twin Layer for network modeling and AI attack detection; Security Layer for threat protection. Using RF Jamming and ToN-IoT datasets, the framework achieves 99% RF jamming detection and 98% composite attack detection while reducing latency by 12%, energy consumption by 15%, and RAM usage by 20%, along with a 6.1% improvement in packet delivery.

### Cybersecurity in EV charging stations

Buedi et al. presents a dataset for EVSE cybersecurity, containing power consumption, network traffic, and host activities under normal and attack conditions^27^. Attacks include DoS, reconnaissance, and V2G interface exploits, collected from a real EVSE using standard protocols. Testing eight ML models, Random Forest and KNN achieved 91% accuracy in binary classification, while Random Forest led multi-class tasks (78.87% accuracy). Ullah et al. propose a privacy-preserving IDS for IoVs, integrating Federated Learning with Deep Swarm Particle Optimization for feature selection^28^. Integration improves deep learning models’ performance, ensuring efficient data analysis in IoV environments. The model achieves 99% accuracy in detecting DoS, spoofing, and benign traffic. Hegde et al. compare centralized vs. decentralized charging networks, showing Ensemble Learning’s superiority for attack detection^29^. Using HPCs and SECCs, their approach enhances intrusion detection. Purohit & Govindarasu develop FL-EVCS, a Federated Learning-based anomaly detection system for EV charging stations^30^. By training local networks at CSMS and sharing model parameters with DSO, the system preserves privacy while achieving 97% accuracy, outperforming traditional ML models.

### Cybersecurity in VANET

Kamel et al. expand the VeReMi dataset^31^ to include DoS, replay, disruptive, and Sybil attacks using the Luxembourg SUMO Traffic scenario. Tejasvi Alladi et al.^32^ introduced a deep learning-based intrusion detection system for IoV networks within Cooperative Intelligent Transportation Systems (C-ITS) using LSTM and CNNs. Their approach includes three models: DLCE-1, a single-stage classifier that identifies normal behavior or misbehavior types; DLCE-2 & DLCE-3, two-stage classifiers that categorize data as usual, fault, or attack. These models, deployed on edge servers with RSUs, analyze vehicle data in real-time using the VeReMi Extension dataset, which includes DoS and replay attacks. DLCE-1 achieves the highest F1 scores (95.58%–96.75%), while DLCE-2 and DLCE-3 offer alternatives.

Hadri et al. use LightGBM to detect Sybil nodes in VANETs via position verification^33^. Using the VeReMi dataset, LightGBM achieves a 0.99 F1-score, outperforming SVM, KNN, RF, and DT through Gradient-based One-Side Sampling and Feature Bundling. Aryal et al. develop a DDoS detection system using an ensemble of six ML models^7^. Random Forest, Decision Tree, and KNN outperform others, with the ensemble reaching 99.83% accuracy in simulated V2V communication. While Random Forest performs best, an ensemble model offers better resilience.

Rukhsar Sultana and colleagues propose a Deep Reinforcement Learning framework for detecting Sybil attacks in VANETs using Deep Q-Networks (DQN)^34^. The model adapts to changing environments without needing large datasets, achieving 96.36% and 95.56% accuracy on two attack datasets. It functions through initial training and real-time adjustment based on network changes such as vehicle speeds. A speed boundary check validates actions. The framework offers a scalable solution for attack detection in VANETs.

Zhao et al. propose distributed event-triggered control to mitigate DoS attacks in vehicle platoons^35^. The research presents a switched system model with event-triggered control and a sampled-data framework, enabling adaptation to normal conditions and DoS disruptions. Using dual-period sampling, the system reduces data transmission by 50% while maintaining safe distances. However, synchronization and scalability are still unexplored. Xiao et al. integrate neural-network adaptive control with data packet processing to counter DoS attacks in V2V networks^36^. Their approach improves resilience against nonlinearities and data losses, although latency impacts need further study.

Sedar et al. propose a deep reinforcement learning (DRL) approach^37^ to counter data poisoning attacks in V2X communication, focusing on label flipping and policy induction. Using Q-Learning, the model trained on the VeReMi dataset achieves 99% accuracy against attacks. Shahid et al.^38^ presents a transfer learning technique using CNN-BiLSTM to detect DoS attacks in V2V networks. The model, pre-trained on KDD19 and CICCSE-IDS datasets with frozen CNN layers and fine-tuned LSTM layers on VeReMi, shows improved attack detection. Sultana et al. introduce LA-DETECTS, a local adaptive misbehavior detection system for VANETs^39^. It addresses false positives through plausibility and consistency checks with Kalman filter-based error estimation to detect data forgery, Sybil, and DoS attacks. The system adjusts parameters dynamically to reduce false alarms and uses weighted decisions for better accuracy. Hampel filters and statistical methods enhance detection, while local operation reduces overhead. However, computational complexity may limit performance in constrained environments.

### Comparison with state-of-the-art

Recent advances in Internet of Things and cyber-physical system security have explored various integrations of blockchain, machine learning, federated learning, and transfer learning to enhance intrusion detection and data privacy. For example, in^40^, have proposed various blockchain-integrated frameworks for the security of data sharing and cyber-defense in distributed environments: a 6G-enabled privacy-preserving IoT healthcare data sharing system; a blockchain framework, with optimized GAO-XGBoost and ECC for intrusion detection and secure data management^41^; and a hybrid blockchain-based architecture for resilient IDS security in IoT deployments^42^. Explainable deep learning models have also been developed for intrusion detection in Industry 5.0 cyber-physical systems^43^, and hybrid blockchain–federated learning approaches have been devised for achieving decentralized and privacy-preserving intrusion detection in IoT networks^44^. Transfer-learning-based IDS models like TL-BILSTM IoT have achieved enhanced intrusion prediction across related IoT environments^45^. Compared with these models, FeXAI offers a lightweight, interpretable, and communication-efficient intrusion detection framework that integrates federated learning, traditional machine learning, and explainable AI, with sensitive data residing at the edge devices. Without blockchain or deep neural architectures, the emphasis of FeXAI is on deployability in resource-constrained smart city subsystems, such as IoV, EVSEs, and VANETs, by focusing on model efficiency, privacy preservation, and transparency.

Although various AI models have been explored for attack detection in IoT applications, a gap remains in classifying attack types, with existing studies showing lower accuracy than binary models. Most approaches focus on isolated IoT security aspects, lacking a unified framework for transport and power management attacks. Our work introduces FeXAI, an Explainable Federated Learning Cybersecurity Framework for smart cities. FeXAI combines Explainable AI, Federated Learning, and Random Forest to evaluating attack classification on datasets collected from Internet of Vehicles, Electric Vehicle chargers, and Vehicular Ad Hoc Networks.

## Proposed methodology

### Data acquisition

This study uses three IoT datasets—CICIoV2024, CICEVSE2024, and the VeReMi Extension. CICIoV2024 focuses on the Internet of Vehicles (IoV), analyzing network traffic between connected vehicles and infrastructure. CICEVSE2024 examines Electric Vehicle Supply Equipment (EVSE), capturing network traffic and activities of EV charging stations during normal and attack scenarios. The VeReMi Extension dataset emphasizes misbehavior detection in Cooperative Intelligent Transport Systems (C-ITS), capturing vehicle data with sensor anomalies and attacks, and serves as a benchmark for vehicular networks.

The CICIoV2024 dataset^1^ was created using a 2019 Ford vehicle to capture realistic traffic and simulate attacks. It records Controller Area Network (CAN) bus traffic, which manages interactions between electronic control units (ECUs) and vehicle parts. The CAN bus operates with High-Speed CAN for time-critical data and Low-Speed CAN for less urgent communications. The dataset includes cyberattack scenarios such as Denial-of-Service (DoS), spoofing, and fuzzing, targeting CAN bus weaknesses. Malicious traffic was produced with custom scripts and can-utils for packet sending and editing. Normal traffic was recorded with candump as a baseline for detecting attacks. The vehicle’s CAN bus was accessed through the OBD-II port, using USB2CAN and Macchina M2 for communication. Analysis was performed with Wireshark and SocketCAN, resulting in a dataset structure that includes arbitration IDs, data fields, and control bits for the Internet of Vehicles (IoV) network.

**Fig. 1:**
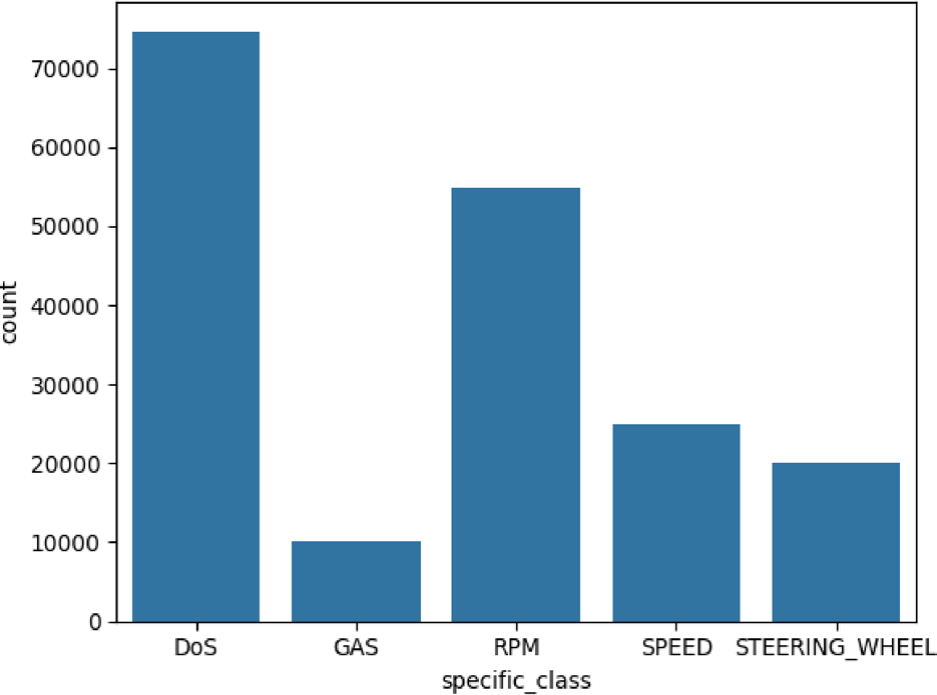
Distribution of attack types in CICIoV2024 dataset.

The class distribution shown in Fig. 1; Table 1 indicates both benign and malicious activities. The Benign class includes 1,223,737 records of normal vehicle communication, serving as a baseline for threat detection. Denial-of-Service (DoS) attacks make up 7,466,300 records, highlighting their importance in disrupting vehicle operations through system flooding.

**Table 1:** Number of instances of each class in the CICOV2024 dataset.

Class	Number of records
Benign	1223737
DoS	7466300
Spoofing (Gas)	9991
Spoofing (RPM)	54900
Spoofing (Speed)	24951
Spoofing (Steering Wheel)	19977

Beyond DoS, the dataset includes various spoofing attack types targeting vehicle parameters. Spoofing (Gas), with 9,991 records, indicates attempts to manipulate fuel data. Spoofing (RPM), with 54,900 records, is the most frequent attack, targeting engine RPM data. Spoofing (Speed) with 24,951 records and Spoofing (Steering Wheel) with 19,977 records show attempts to alter vehicle control data, posing safety risks. The frequencies highlight the need for security measures, especially against RPM data manipulation.

**Fig. 2:**
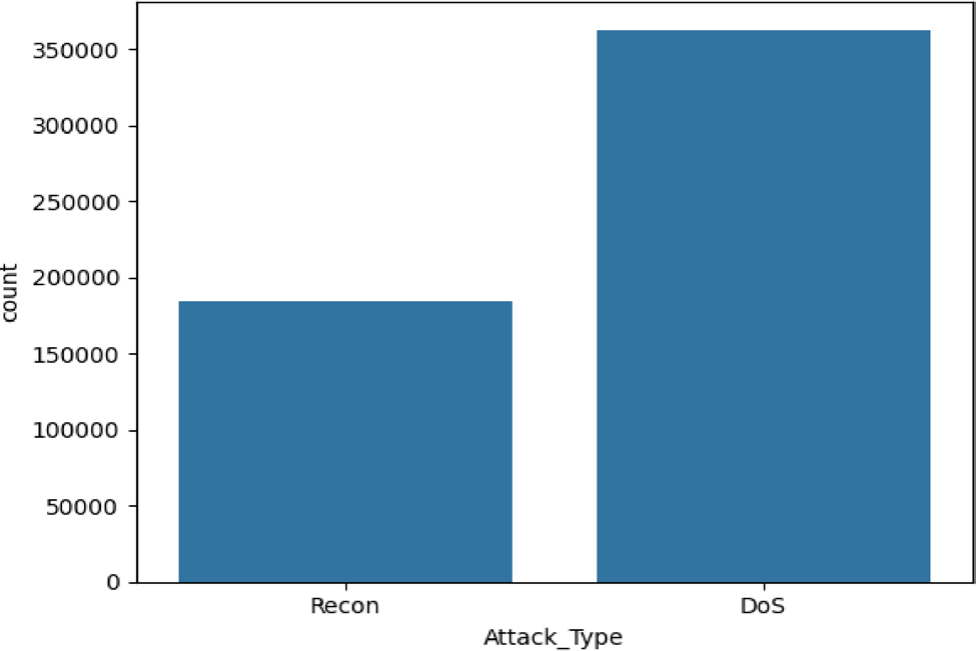
Distribution of attack types in CICEVSE2024 dataset.

The CICEVSE2024 dataset^27^ analyzes Electric Vehicle Supply Equipment (EVSE) behavior during normal operations and cyberattacks. It includes both benign activities and malicious events like Denial-of-Service (DoS) and Reconnaissance attacks. The dataset captures network traffic from ISO15118 and the Open Charge Point Protocol (OCPP), which facilitate communication between EVSEs and the Charging Station Management System (CSMS) for authentication, monitoring, and transaction management. Using Wireshark and TCPdump, the dataset examines packets exchanged during charging sessions, providing insights into EVSE-CSMS interactions. The attack distribution is shown in Fig. 2.

**Table 2:** Number of instances of each class in the CICEVSE2024 dataset.

Class	Number of records
DoS	363152
Recon	184620
Benign	82

**Fig. 3:**
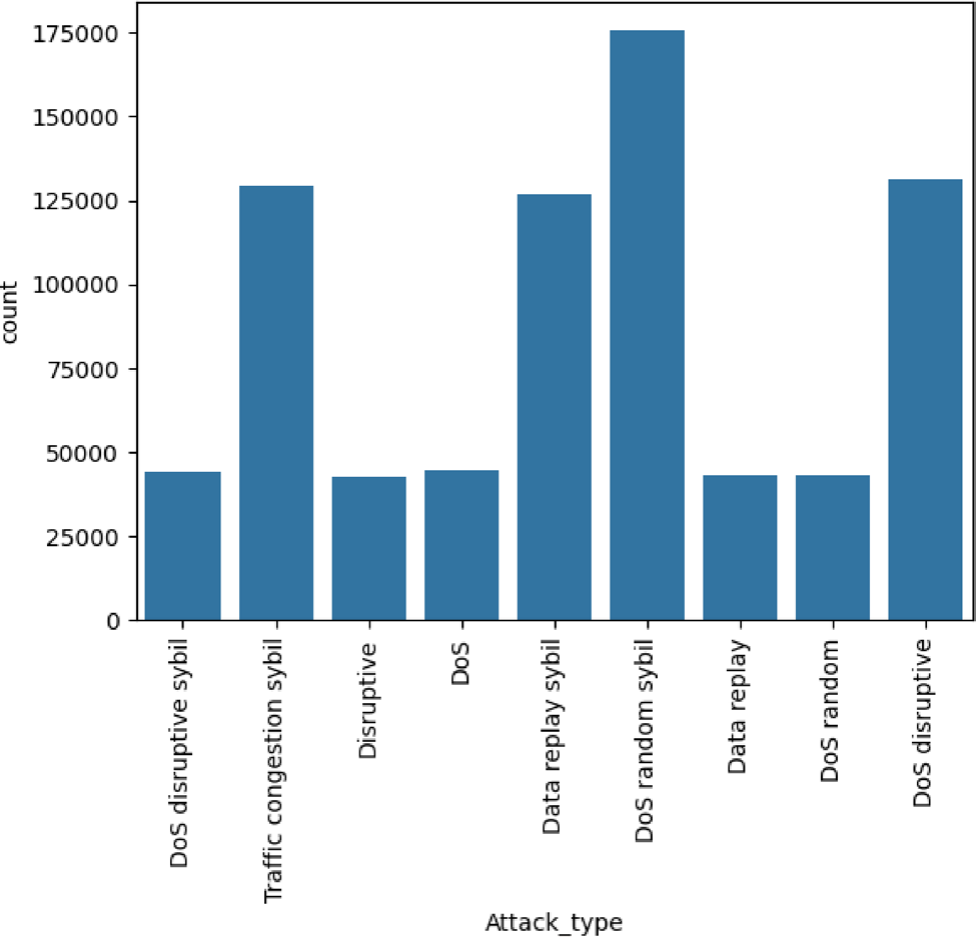
Distribution of attack types in VeReMi extension dataset.

As shown in Table 2, CICEVSE2024 contains only 82 Benign records, highlighting malicious activity simulation over normal behavior. The DoS class has 363,152 records, making it the most common and focusing on attacks that disrupt operations. The dataset includes 184,620 Recon records for reconnaissance activities and is designed to test system resilience against probing and cyberattacks. The VeReMi extension dataset^31^ was created using the Framework for Misbehaviour Detection (F2MD), which combines the OMNeT + + network simulator with the SUMO traffic simulation tool. It uses a subsection of the Luxembourg SUMO Traffic scenario, simulating vehicular behavior over a 1.61 km² area with a peak density of 67.4 vehicles per km². The dataset includes sensor error models that introduce inaccuracies in vehicle data, such as position, velocity, acceleration, and heading errors, to simulate GPS limitations and environmental interferences. It features both unintentional malfunctions and intentional cyberattacks, including Denial of Service (DoS), data replay, and traffic congestion manipulation, enabling the study of vehicular misbehavior. For this research, the focus will be on malicious attacks, with the distribution shown in Fig. 3.

**Table 3:** Number of instances of each class in the VeReMi extension dataset.

Class	Number of records
Normal behaviour	1900539
DoS	44337
Data replay	43264
Disruptive	42790
DoS random	43118
DoS random sybil	175391
DoS disruptive	131305
Traffic congestion sybil	129270
Data replay sybil	126724
DoS disruptive sybil	44310

Table 3 shows the VeReMi Extension dataset, which includes various attack types centered on Denial-of-Service (DoS) and traffic-related threats. The Normal Behavior class, with 1,900,539 records, acts as a baseline for detecting cyberattack deviations. The dataset features DoS attack variants such as DoS random, DoS random Sybil, and DoS disruptive Sybil. These demonstrate different methods used to overload network resources and disrupt vehicular communication. Other attack categories like Data Replay, Disruptive, and Traffic Congestion Sybil represent specific actions aimed at manipulating network traffic and compromising system integrity. The number of attack records emphasizes the dataset’s focus on simulating network threats and provides insights into both normal and malicious vehicular behavior.

### Data preprocessing

A series of techniques were used for data cleaning and preprocessing. First, files from each class were merged into one dataset to ensure consistency. Also, some files were missing the label or category column, which was reconstructed based on the file names to keep proper classification. Then, null values were identified and removed using a structured method, following Eq. (1) to eliminate incomplete data and improve dataset integrity.

1 $$\:{X}_{non-null}=X\setminus\:\left\{{X}_{missing}\right\}$$

Non-numeric data was converted to numerical categorical values using LabelEncoder from scikit-learn library. This transformation encoded string-based features for processing, following Eq. (2).

2 $$For~x~ \in ~\left\{ {C_{1} ,~C_{2} ,~ \ldots ,C_{k} } \right\},~~~~x_{{encoded}} = \left\{ {0,1, \ldots ,k} \right\}$$

Features were analyzed for potential linear relationships through correlation. High correlations can introduce multicollinearity, which may distort model coefficients and impact performance. A correlation matrix was computed using the Pearson correlation coefficient, as defined in Eq. (3).

3 $$\:{r}_{XY}=\:\frac{\sum\:_{i=1}^{n}\left({X}_{i}-\stackrel{-}{X}\right)\left({Y}_{i}-\stackrel{-}{Y}\right)}{\sqrt{\sum\:_{i=1}^{n}{\left({X}_{i}-\stackrel{-}{X}\right)}^{2}\sum\:_{i=1}^{n}{\left({Y}_{i}-\stackrel{-}{Y}\right)}^{2}}}$$

**Fig. 4:**
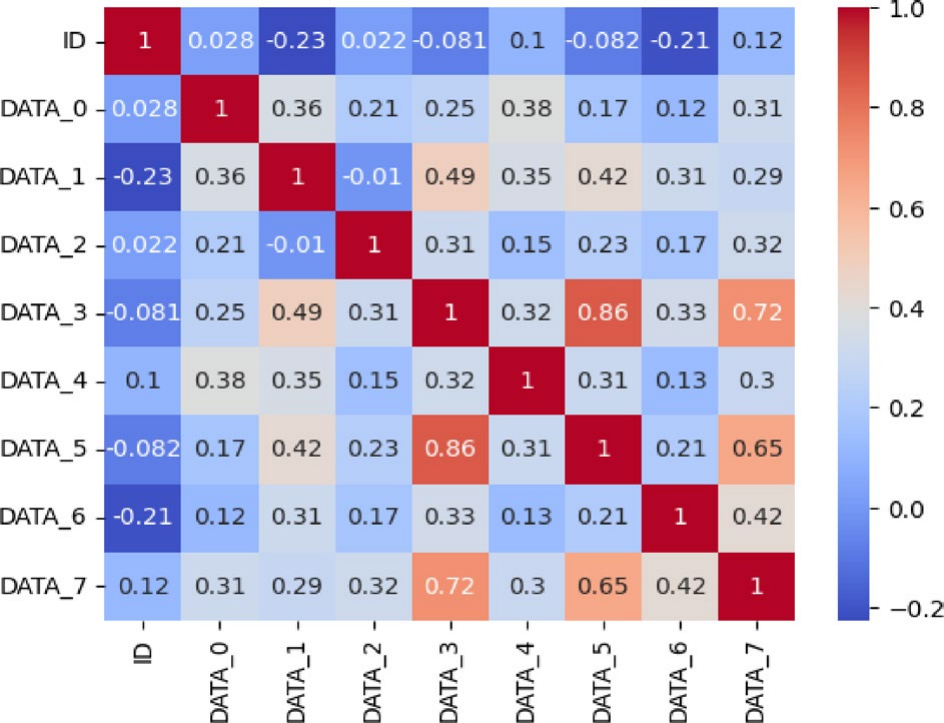
CICIoV2024 dataset correlation matrix.

**Fig. 5:**
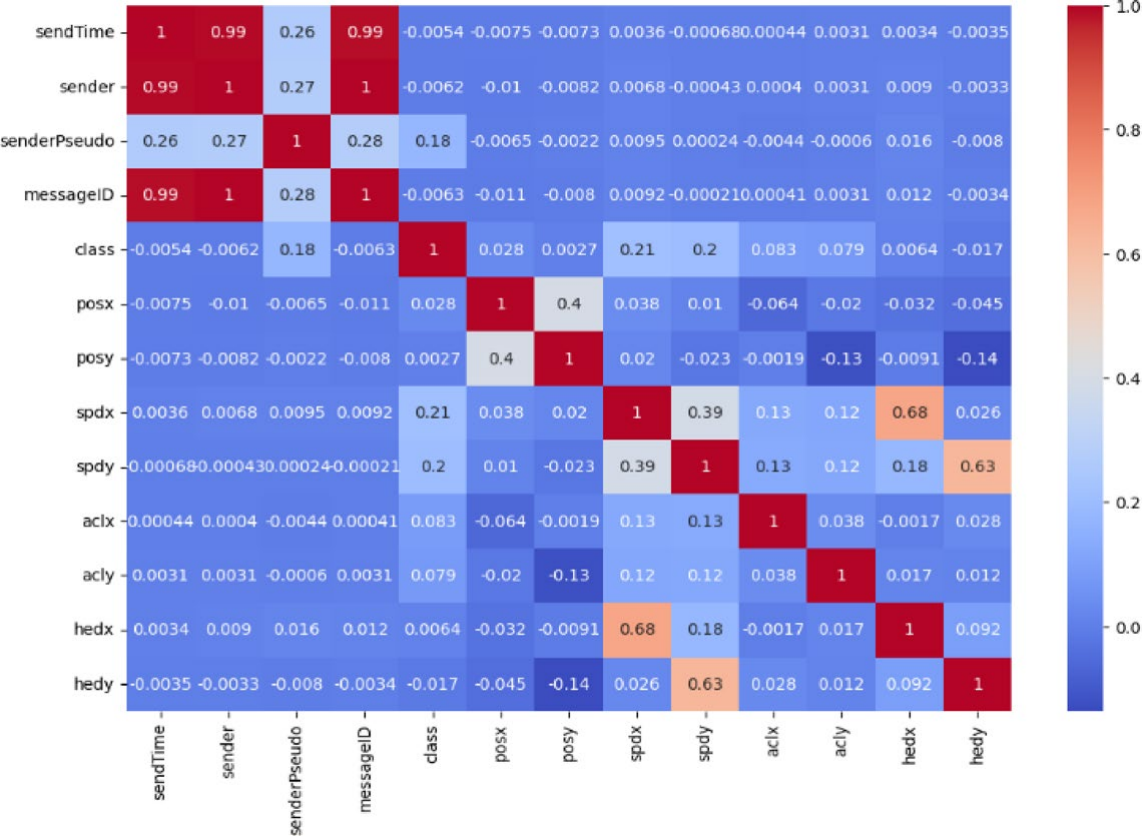
VeReMi extension dataset correlation matrix.

Figures 4 and 5 display correlation matrices for the CICIoV2024 and VeReMi datasets. In Fig. 4, there is a strong correlation between DATA_3 and DATA_5, leading to the inclusion of one in the final feature set. Similarly, in Fig. 5, sendTime shows a high correlation with the sender and messageID fields. To streamline features, sendTime is retained for model training.

**Fig. 6:**
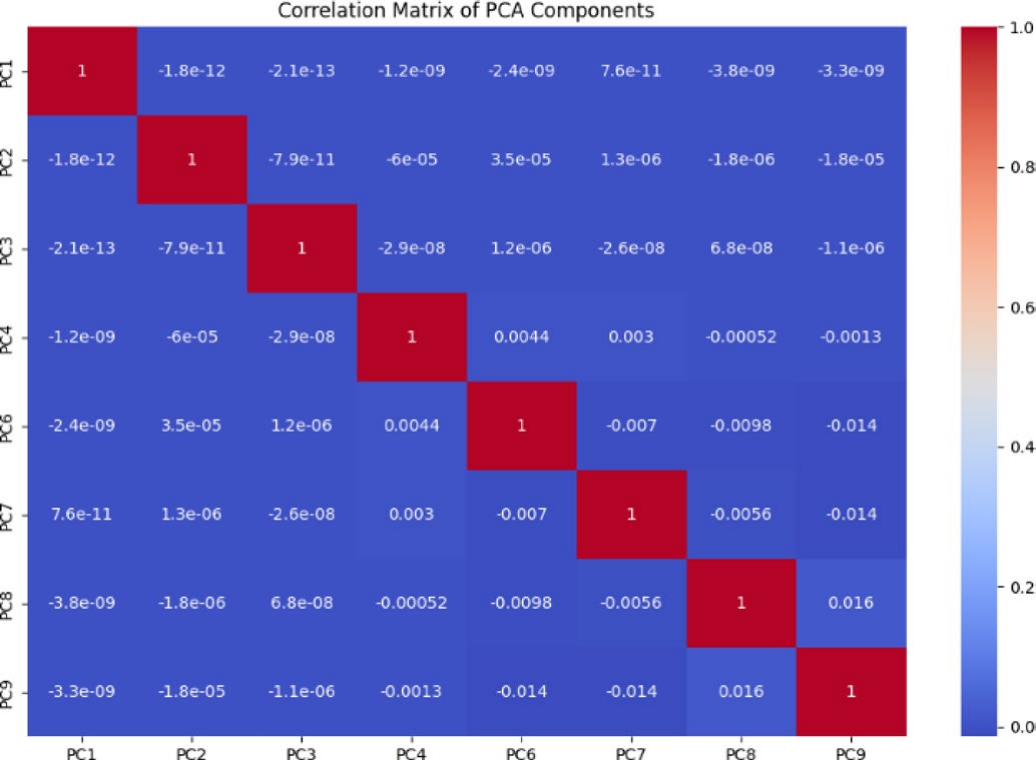
CICEVSE2024 PC correlation matrix.

The CICEVSE2024 dataset contains numerous features, which makes visualization and model training difficult. Principal Component Analysis (PCA) is used to reduce the number of dimensions by transforming correlated features into uncorrelated principal components (PCs), calculated using Eq. (4). These components preserve the maximum variance while simplifying the data. The correlation matrix of the principal components is shown in Fig. 6.

4 $$\:Cv=\:\lambda\:v$$

To address class imbalance, SMOTE was used to generate synthetic samples with Eq. (5), enhancing classification by reducing major class bias. This technique helps in situations where the minority class is underrepresented. However, for large datasets, SMOTE increases computational load and might lead to overfitting.

5 $$\:{x}_{synthetic}={x}_{1}+\:\lambda\:({x}_{2}-\:{x}_{1})$$

Another approach examined was undersampling, which reduces instances in the majority class to balance the dataset by removing redundant samples. However, undersampling risks losing information and underfitting, as valuable patterns from the majority class may be discarded.

To address class imbalance, this study uses class_weight=’balanced’ to compute weights for each class based on frequency using Eq. (6) during training. This helps reduce bias toward the majority class and ensures better consideration of the minority class while preventing overfitting.

6 $$\:{w}_{i}=\:\frac{{n}_{samples}}{{n}_{classes}\times\:{n}_{samples\:of\:class\:i}}$$

**Table 4:** Ablation study on CICIoV2024 dataset.

Method	Accuracy	Precision	Recall	F1-score
Class weighting (balanced)	**0.9964**	**0.9970**	**0.9964**	**0.9965**
SMOTE oversampling	0.9964	0.9970	0.9964	0.9965
ADASYN oversampling	*NA*	*NA*	*NA*	*NA*
No rebalancing	0.9964	0.9967	0.9964	0.9963

The values highlighted in bold are the best-performing results for the respective evaluation metrics.

The ablation study, represented in Table 4, presents the finding that class weighting performs equally well to SMOTE in terms of accuracy (0.9964), precision (0.9970), recall (0.9964), and F1-score (0.9965), proving that, SMOTE oversampling does not have any measurable advantage for this dataset. This is anticipated considering the large sample sizes in most classes, as these let the Random Forest models learn balanced decision boundaries even under moderate class imbalance. ADASYN could not be applied due to insufficient minority-class neighbor density, which is a known limitation when dealing with extremely sparse categories. The baseline model, trained without any balancing of imbalanced data, resulted in somewhat lower precision and F1-score, which confirms the view that at least some form of balancing is required. Class weighting is thus empirically validated as an optimal and most efficient imbalance mitigation strategy for the proposed FeXAI framework.

### Types of network attacks in the IoT domain

This study examines network attacks on IoT systems within the Internet of Vehicles (IoV) and Electric Vehicle Supply Equipment (EVSE). A brief overview of each attack type is provided below:

#### Denial of service (DoS) attack

A DoS attack disrupts system functions by overwhelming it with too many requests, depleting resources such as bandwidth and processing power.

- In EVSE, a DoS attack could disrupt communication between the EV and CSMS, rendering charging stations unresponsive to legitimate users.
- In IoV, a DoS attack could target V2I communication, disrupting traffic control and vehicle services.

#### Spoofing attack

Spoofing happens when an attacker impersonates a legitimate entity to gain unauthorized access or manipulate the system.

- In EVSE, attackers could impersonate EVs or charging stations to gain unauthorized access, manipulate billing, or modify communication data.
- In IoV, spoofing can let malicious entities impersonate vehicles or infrastructure, causing false signals and risking road safety.

#### Reconnaissance (Recon) attack

A reconnaissance attack involves collecting intelligence about a target system to find vulnerabilities that could be exploited in future attacks.

- In EVSE, attackers might scan for vulnerabilities in communication protocols such as ISO15118 or OCPP, as well as in power management systems and network infrastructures, which could later be targeted in DoS or spoofing attacks.
- In IoV, reconnaissance attacks may be used to identify weaknesses in vehicle communication protocols, potentially causing traffic disruptions or enabling more sophisticated cyberattacks.

#### Distributed denial of service (DDoS) attack

A DDoS attack is a complex DoS where multiple compromised systems flood the target with traffic from various sources, making mitigation more difficult.

- In EVSE, a DDoS attack could disrupt charging station networks by overwhelming servers.
- In IoV, DDoS attacks can disrupt V2V and V2I communications, leading to navigation failures and traffic delays.

#### Sybil attack

A Sybil attack occurs when an adversary creates multiple fake nodes in a network to manipulate operations.

- In VANETs, attackers can create fake vehicles to spread false traffic information, causing rerouting or congestion. Sybil attacks in IoV can overwhelm systems with fake identities, impacting routing and EV charging resources.

#### Replay attack

A Replay attack occurs when an attacker resend captured data to trick recipients into believing it’s legitimate.

- In VANETs, attackers could replay collision warnings or traffic alerts, leading vehicles to respond to outdated information, which may result in accidents.
- In IoV, replayed messages could disrupt routing, traffic signals, or emergency systems.
- In EV charging networks, attackers could impersonate stations by replaying authentication messages to steal electricity or gain unauthorized access.

These attacks present serious threats to system functionality and user safety. As IoT systems grow more interconnected, strong security measures become essential. Advanced detection methods, secure protocols, and proactive strategies will help maintain IoT system resilience. Implementing robust security is vital to reducing vulnerabilities and ensuring system reliability.

### Machine learning

To develop the proposed framework, multiple machine learning models were tested to find the most effective method for attack classification. The techniques explored are outlined below:

#### Logistic regression

Logistic Regression is a statistical model used for binary classification, predicting class probabilities. For multiclass problems, it employs one-vs-rest or multinomial methods. Training involves minimizing a cost function, usually log-loss, as shown in Eq. (7).

7 $$\:J\left(\theta\:\right)=\:-\frac{1}{m}\sum\:_{i=1}^{m}\sum\:_{c=1}^{C}{y}_{ic}\mathrm{log}\left({h}_{\theta\:}\right({x}_{i}\left)\right).\:{w}_{c}$$

Class weights increase the penalty for misclassifying minority instances during training, prompting the model to focus on these examples and adjust decision boundaries. The optimization process uses a modified cost function with class weights, ensuring that logistic regression minimizes error while addressing imbalance. This enhances the model’s sensitivity to minority classes, boosting their prediction accuracy while preserving majority class performance.

#### Naïve Bayes

Naïve Bayes is a probabilistic algorithm based on Bayes’ Theorem that assumes feature independence given class labels. In multiclass classification, it estimates class conditional probabilities using Gaussian, Multinomial, or Bernoulli distributions. To handle class imbalance, the model adjusts priors to prevent bias toward the majority class, as shown in Eq. (8).

8 $$\:P\left({C}_{k}|x\right)\:\alpha\:\:P\left({C}_{k}\right)\:.\:\prod\:_{i=1}^{n}P\left({x}_{i}\right|{C}_{k})\:$$

#### Nearest neighbours

K-Nearest Neighbors (KNN) is a non-parametric algorithm that predicts outcomes by utilizing the training dataset instead of building a model. It assumes that similar data points have similar results.

KNN calculates distances between data points to identify the nearest neighbors, usually using Euclidean distance as defined in Eq. (9).

9 $$\:d\left(p,q\right)=\:\sqrt{\sum\:_{i=1}^{n}{({p}_{i}-\:{q}_{i})}^{2}}$$

#### Random forest

Random Forest is an ensemble learning algorithm that constructs multiple decision trees and predicts the final class through majority voting. It is known for its robustness, ability to handle high-dimensional data, and strong generalization. As a bagging technique, Random Forest trains models on different subsets of data and combines their predictions to improve accuracy and reduce overfitting. The algorithm follows these steps:

*Bootstrap Sampling*: Random Forest creates data subsets by bootstrapping, where samples are randomly chosen with replacement. This ensures trees train on different datasets, increasing diversity and decreasing overfitting.

*Random Feature Selection*: At each node split, the algorithm chooses a random subset of features, decreasing correlation between trees and enhancing generalization.

*Independent Tree Training*: Each tree is trained on its bootstrapped dataset and feature subset without pruning.

*Majority Voting for Prediction*: The final prediction combines the outputs of individual trees. For classification, the class that appears most often among trees becomes the final result.

Random Forest uses Gini Impurity as a criterion for splitting nodes, measuring class impurity at each decision point. The Gini Impurity for a node t is mathematically defined in Eq. (10).

10 $$\:G\left(t\right)=1-\:\sum\:_{i=1}^{C}{P}_{i}^{2}$$

### Federated learning

Federated learning is an advanced machine learning approach that enables collaborative model training across multiple decentralized devices or servers while maintaining data privacy. Unlike traditional centralized methods, where data is gathered and processed in a single location, federated learning allows models to be developed without transferring sensitive data to a central server, making it especially suitable for privacy-sensitive applications. The main idea focuses on decentralized data storage and distributed model training.

In this approach, participants such as IoT devices locally train their models on their respective datasets. Instead of sharing raw data, these participants compute model updates, such as gradients or weights, which are then securely transmitted to a central server. The central server aggregates these updates using techniques like Federated Averaging (FedAvg) to improve a global model.

This process is repeated iteratively, with the updated global model being sent back to participants for further local training until the convergence occurs. By enabling privacy-preserving collaborative learning, federated learning is especially useful for applications in healthcare, finance, and IoT networks, where maintaining data confidentiality is essential. The structured steps of this learning process are described in Algorithm 1.

**Algorithm 1:**
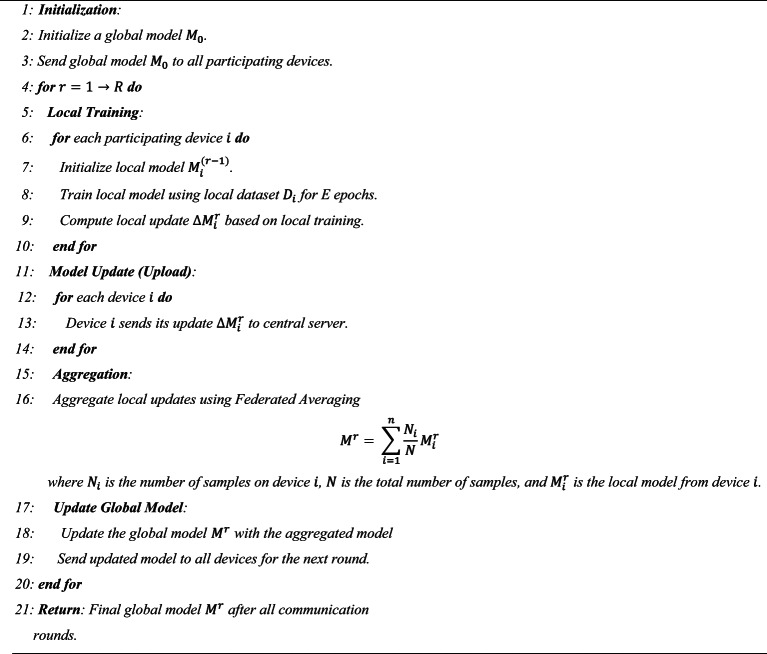
Federated learning algorithm.

### Explainable AI

Explainable AI (XAI) includes methods that improve the transparency and interpretability of AI systems, making their decisions easier to understand. By boosting trust and fairness, XAI encourages wider AI use across various fields. Many AI models act as black boxes, producing predictions without showing their reasoning. XAI solves this by offering insights into decision-making processes, helping users understand the logic behind outcomes. This transparency builds trust and makes it easier to spot biases in AI models, supporting fairer decisions. XAI methods also assist in debugging models by identifying key features that influence decisions.

One key XAI method is SHAP (SHapley Additive exPlanations), which interprets machine learning models using feature importance scores. SHAP values originate from cooperative game theory’s Shapley values, with each feature considered a “player” contributing to predictions. The Shapley value is computed by averaging its marginal contribution across different feature subsets. Calculating exact Shapley values requires assessing all possible feature combinations, leading to exponential complexity as the number of features grows. This makes it computationally costly for large-scale models. For complex models, the general SHAP algorithm becomes inefficient because of the necessary evaluations. The Shapley value for a feature is mathematically defined in Eq. (11).

11 $$\phi \left( {f_{i} } \right) = ~\mathop \sum \limits_{{S \subseteq F\backslash f_{i} }} \frac{{\left| S \right|!\left( {\left| F \right| - \left| S \right| - 1} \right)!}}{{\left| F \right|!}}\left[ {v\left( {S \cup ~f_{i} } \right) - v\left( S \right)} \right]~$$

TreeSHAP is an improved version of SHAP designed for tree-based models like Random Forests. Unlike traditional SHAP, which calculates all feature subsets, TreeSHAP uses the hierarchical structure of decision trees to compute Shapley values more efficiently. This makes it practical to determine feature attributions for large ensembles. TreeSHAP takes advantage of how tree models make predictions through decision paths based on feature splits. It finds feature contributions by analyzing these paths and assessing how features impact the model’s output. By combining these contributions across different paths, TreeSHAP decreases computational demands while keeping accuracy high. The contribution of a feature along a decision path is defined in Eq. (12).

12 $$Contribution\left( {f_{i} } \right) = ~\frac{{p\left( {leaf} \right)}}{{2^{{\left| {path} \right|}} }}\left[ {\hat{y}_{{with}} - ~\hat{y}_{{without}} } \right]$$

For ensemble models like Random Forests, TreeSHAP determines the Shapley value by averaging each feature’s contributions across all trees in the ensemble, as shown in Eq. (13).

13 $$\:\:\varphi\:\left({f}_{i}\right)=\:\frac{1}{m}\sum\:_{j=1}^{m}{\varphi\:}_{j}\left({f}_{i}\right)$$

### Proposed framework

**Fig. 7:**
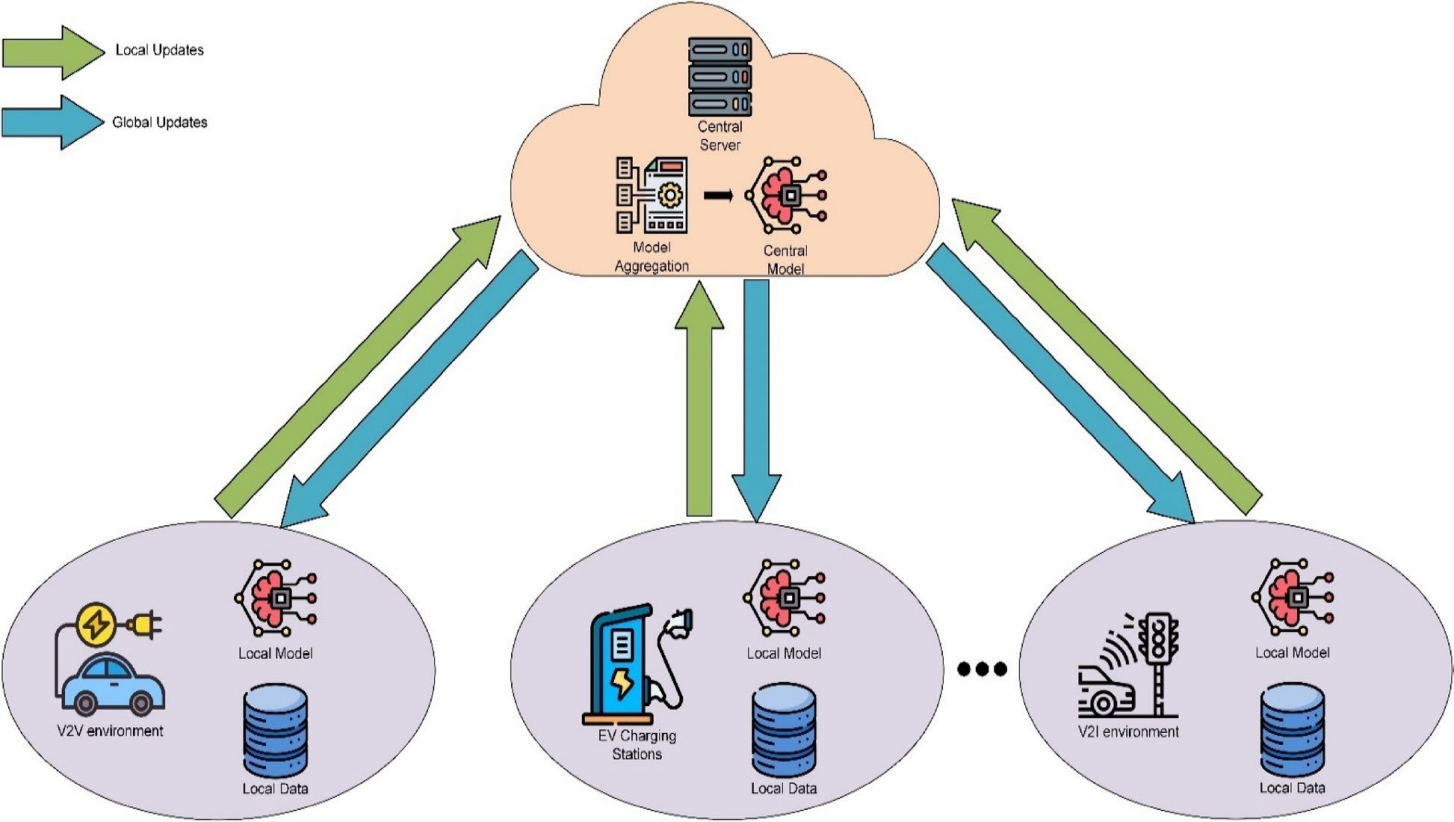
Architecture of FeXAI framework.

The FeXAI framework aims to improve cybersecurity in smart cities by combining Explainable Artificial Intelligence (XAI), Federated Learning, and Random Forest algorithms. FeXAI targets the detection and mitigation of cyber threats in areas like the Internet of Vehicles (IoV), Electric Vehicle (EV) charging systems, and Vehicular Ad Hoc Networks (VANETs). This unified strategy boosts intrusion detection and classification, ensuring stronger security. By using adaptive learning, FeXAI enhances the protection of critical smart city infrastructure. The framework’s design, shown in Fig. 7, includes multiple local edge devices that store and process local data to train individual models. These models send parameter updates to a central server, which combines them to form a global model. The global model is then sent back to the edge devices for local updates. This cycle repeats over multiple training rounds, enabling continuous learning and improvements across distributed nodes. FeXAI employs Federated Learning to enable decentralized attack detection while maintaining data privacy. By spreading the learning process across various nodes, distributed across multiple simulated clients, reflecting privacy and data-separation constraints commonly seen in IoV and smart grid environments. This decentralized method strengthens security and promotes collaboration among interconnected systems.

To ensure reproducibility and allow for a fair comparison with existing intrusion detection methods, the complete configuration of the Random Forest classifier used in FeXAI is presented. For the RF model, it was trained with 10 decision trees (n_estimators = 10), a maximum depth of 40, a minimum split sample size of 2, and a minimum leaf sample size of 1. For impurity criteria, Gini impurity was used for both the IoV and VANET datasets, whereas entropy was used for the EVSE dataset. Feature sampling followed the default sqrt strategy. Class imbalance was addressed using class_weight = ‘balanced’. All experiments were conducted on CPU and in a federated setting where each round of communication took approximately 4.88 s for all three clients combined, including both model training and communication overhead.

To enhance performance and interpretability, FeXAI uses Random Forest, a highly effective machine learning model known for its robustness, accuracy, and low computational requirements. Its capacity to handle imbalanced data and multi-class attack detection is further improved by methods such as weighted training, making it particularly suitable for resource-limited environments in smart cities. A key feature of FeXAI is its integration of Explainable AI (XAI) techniques, which offer transparency in decision-making. By providing clear insights into model predictions, stakeholders can better understand detected threats, refine response strategies, and build trust in the system. The framework’s operational workflow is detailed in Algorithm 2.

**Algorithm 2:**
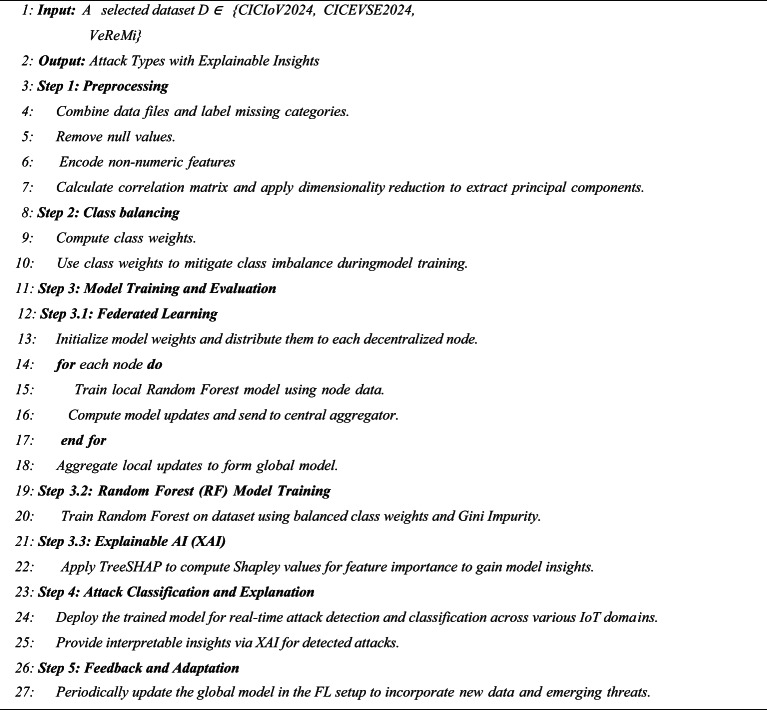
Proposed FeXAI framework algorithm.

FeXAI is built on a modular architecture, allowing easy integration with various urban infrastructures while ensuring scalability and adaptability to new technologies. By incorporating Federated Learning, Random Forest, and Explainable AI, the framework overcomes the limits of traditional intrusion detection systems. It offers a scalable, privacy-focused, and interpretable cybersecurity solution, making it well-suited for securing smart city environments.

## Results and discussion

The performance of each model was assessed using the following evaluation metrics:

- *Accuracy*: Defined in Eq. (14), accuracy represents the proportion of correctly classified instances relative to the total number of instances in the dataset.

14 $$\:Accuracy=\frac{True\:Postives+True\:Negatives}{Total\:Instances}$$

- *Precision*: As outlined in Eq. (15), precision quantifies the proportion of positive predictions that are actually correct, indicating the model’s reliability in identifying positive cases.

15 $$\:Precision=\frac{True\:Postives}{True\:Positives+False\:Positives}$$

- *Recall*: Defined in Eq. (16), recall measures the proportion of actual positive instances that the model successfully identifies, reflecting its ability to detect true positives.

16 $$\:Recall=\frac{True\:Postives}{True\:Positives+False\:Negatives}$$

- *F1 Score*: Given in Eq. (17), the F1 score is the harmonic mean of precision and recall, providing a balanced measure that accounts for both false positives and false negatives.

17 $$\:F1\:score=\frac{2\:\times\:\:precision\:\times\:\:recall}{precision+recall}$$

### Performance metrics analysis

**Table 5:** Results of ML algorithms on CICIOV2024 dataset.

Model	Accuracy	Precision	Recall	F1 score
LR	88%	47%	42%	43%
Naïve Bayes	69%	60%	89%	64%
KNN	**99.5%**	**99%**	97%	97%
RF	**99.9%**	97%	**98%**	**98%**

The values highlighted in bold are the best-performing results for the respective evaluation metrics.

**Fig. 8:**
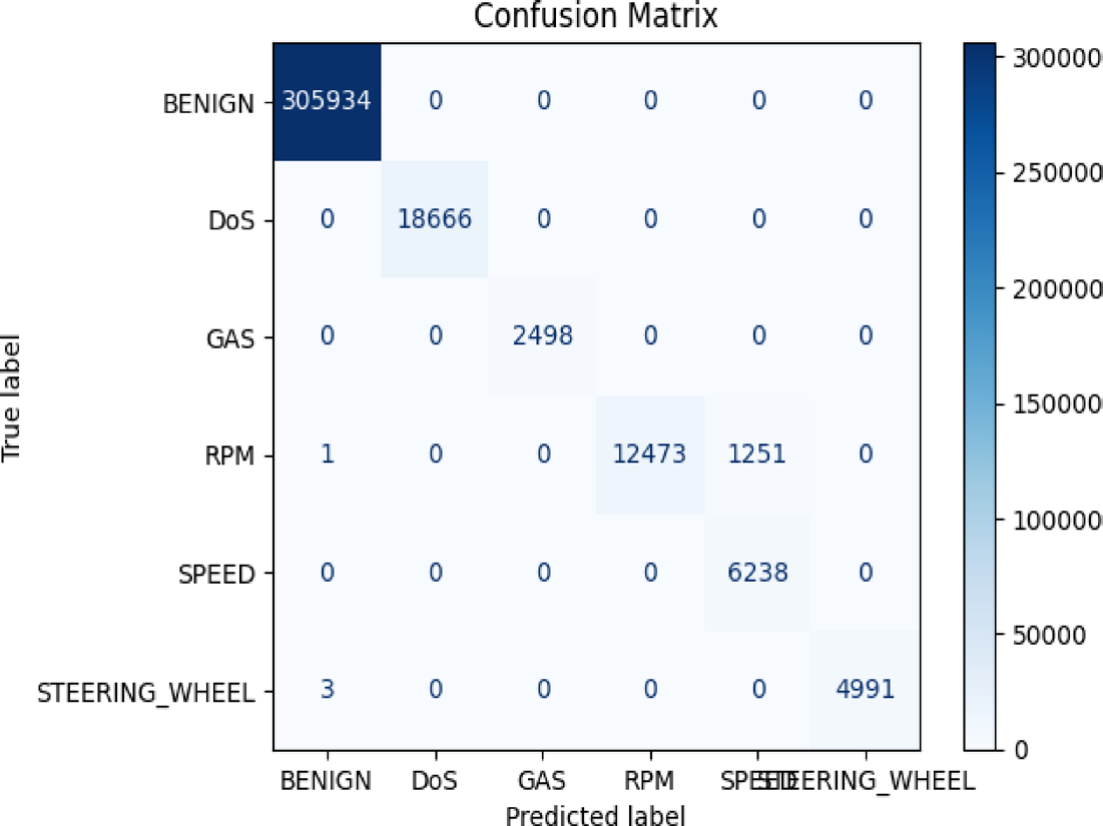
Confusion matrix for Random Forest for CICIoV2024.

Table 5 shows the performance results of different algorithms tested on the CICIoV2024 dataset. Models such as Logistic Regression and Naïve Bayes perform poorly, while Random Forest significantly outperforms other models with an impressive F1-score of 0.98. Although KNN has accuracy and precision similar to Random Forest, its higher computational cost makes it less suitable for real-time use. Figure 8 displays the confusion matrix for the Random Forest classifier, which accurately classifies Benign, DoS, Spoofing Gas, and Spoofing Speed attack classes with perfect identification. The Spoofing Steering Wheel class is also well-classified, with only a few misclassifications. However, the model struggles slightly with Spoofing RPM attacks, with some instances misclassified as Spoofing Speed. Table 6 presents the experimental results on the CICEVSE2024 dataset. Again, Logistic Regression and Naïve Bayes perform poorly, while KNN shows slight improvement and remains consistent with the previous models. Nevertheless, Random Forest provides the best results, achieving a classification accuracy of 99.9%. Table 7 shows the performance metrics for various models on the VeReMi extension dataset, with Logistic Regression performing poorly and Naïve Bayes reaching moderate accuracy.

**Table 6:** Results of ML algorithms on CICEVSE2024 dataset.

Model	Accuracy	Precision	Recall	F1 score
LR	46%	34%	34%	31%
Naïve Bayes	61%	23%	64%	26%
KNN	80.4%	81.56%	80.46%	80.1%
RF	**99.4%**	**98.25%**	**98.2%**	**98.6%**

The values highlighted in bold are the best-performing results for the respective evaluation metrics.

**Table 7:** Results of ML algorithms onVeReMi dataset.

Model	Accuracy	Precision	Recall	F1 score
LR	16%	17%	22%	13%
Naïve Bayes	72%	57%	72%	62%
KNN	99%	98%	96%	98%
RF	**99.9%**	**98.75%**	**97.3%**	**99.4%**

The values highlighted in bold are the best-performing results for the respective evaluation metrics.

**Fig. 9:**
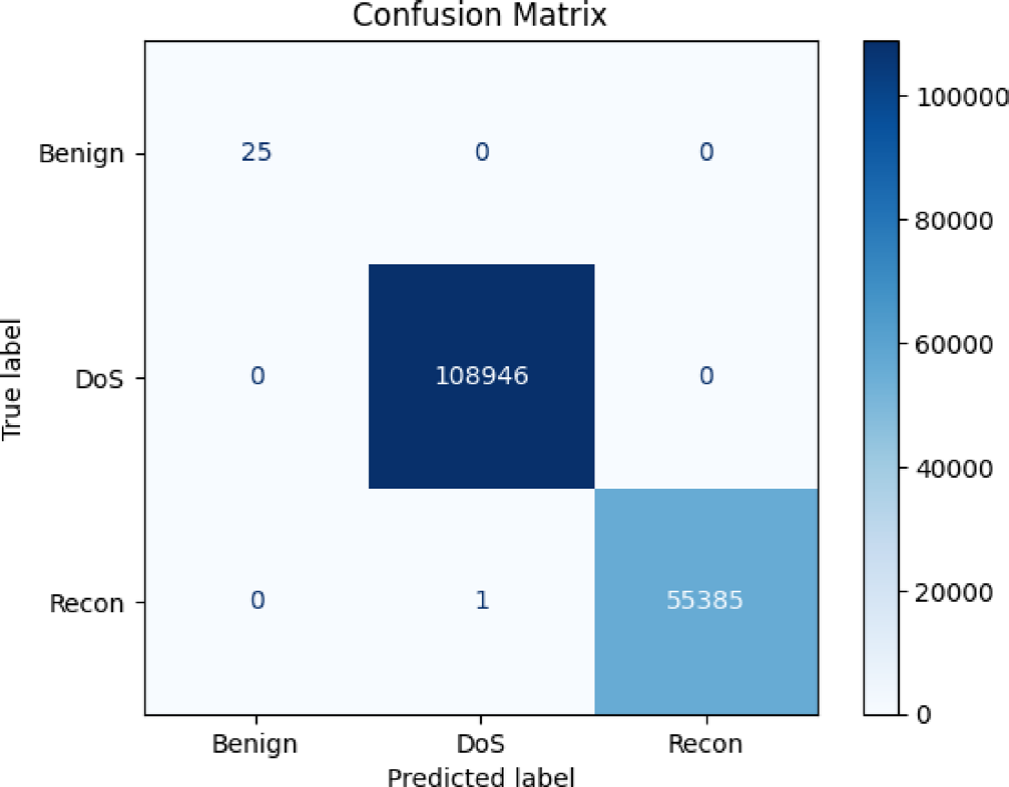
Confusion matrix for Random Forest for CICEVSE2024.

KNN performs well in terms of accuracy and precision, although its recall is slightly lower. Random Forest outperforms all other models, achieving a perfect F1-score of 1.00. Figure 9 shows the confusion matrix for the Random Forest Classifier on the CICEVSE2024 dataset. The Benign and DoS classes are perfectly classified, while the Recon class is also identified with high accuracy, except for a single record misclassified as a DoS attack. Given the dataset size of 55,385 records, this minor misclassification is negligible.

**Fig. 10:**
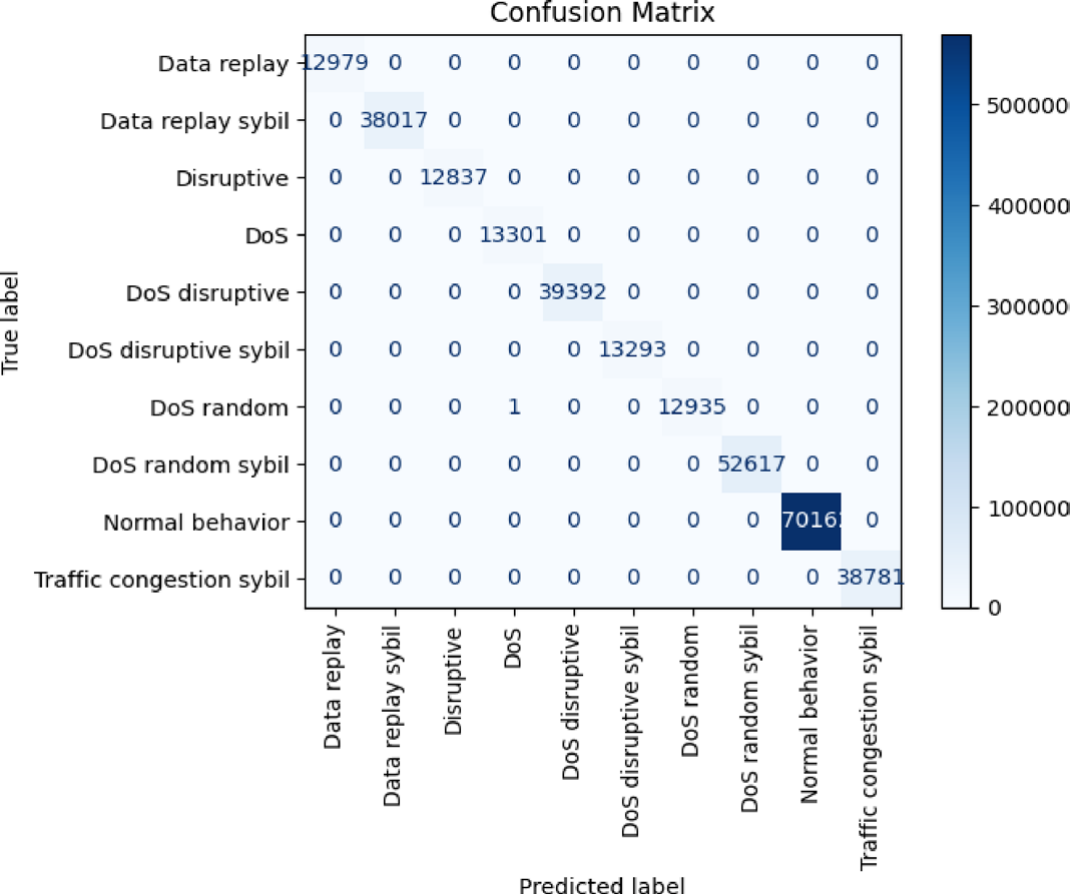
Confusion matrix for Random Forest for VeReMi extension dataset.

Figure 10 shows the confusion matrix for the Random Forest model on the VeReMi extension dataset, indicating that all attack types have been correctly classified. The model effectively distinguishes between different attack categories, including DoS, Sybil, and Replay, along with their subcategories. To verify the model’s reliability and prevent overfitting, its performance was validated using 5-fold cross-validation. The dataset was split into five subsets, with four used for training and one for testing in each round. The final results reported are the average performance across all rounds. Table 8 compares the results from existing literature with those from the current research, focusing on two key metrics: accuracy and F1 score. The analysis shows that the proposed framework outperforms even complex architectures that involve neural networks and meta-heuristic algorithms. To ensure the results are reliable and not due to overfitting, cross-validation was repeated, further confirming the framework’s effectiveness. Also, since the proposed method uses an ensemble machine learning approach, it greatly reduces computational power and memory needs compared to previous work, making it a more efficient and practical solution. Given the limited computing power of potential edge deployments, a federated version of the Random Forest model was evaluated on CICIoV2024 to demonstrate the feasibility of privacy-preserving training.

**Table 8:** Comparisons of Results between existing research and proposed methodology.

Dataset	Methodology	Accuracy	F1 score
CICIoV2024	Random Forest^1^	96%	78%
	Cascaded Long Short-Term Memory [LSTM] and Naive Bayes classifier^2^	99.7%	-
	Genetic Algorithm and Random Forest^23^	99.64%	97.45%
	Deep Convolutional Factorization Machine^24^	91.8%	92.3%
	Feature Fusion and Stacking-based IDS^25^	99.2%	97.3%
	**FeXAI (Proposed methodology)**	**99.9%**	**98.6%**
CICEVSE2024	Support Vector Machine^27^	94.12%	94.77%
	Federated Learning using Deep Neural Network with Deep Swarm Particle Optimization^28^	99.29%	-
	Random Forest^29^	99.2%	95%
	**FeXAI (Proposed methodology)**	**99.5%**	**98.2%**
VeReMi	Deep Reinforcement Learning^37^	99%	98.5%
	Rule-based plausibility and consistency checks^39^	91.54%	90.63%
	Transfer Learning using CNN-BiLSTM^38^	97.8%	97.3%
	**FeXAI (Proposed methodology)**	**99.9%**	**99.4%**

The values highlighted in bold are the best-performing results for the respective evaluation metrics.

**Table 9:** Results of federated learning after two iterations.

Metric	Server	Client 1	Client 2	Client 3
Accuracy	99.66%	99.64%	99.68%	99.65%
Precision	99.71%	99.7%	99.73%	99.7%
Recall	99.66%	99.64%	99.68%	99.65%
F1 score	99.66%	99.64%	99.69%	99.65%

Table 9 shows the results of this approach across the server and three clients. Each client trains and evaluates a local model using its private dataset, receives the global model from the server, updates it with local data, and sends the revised model parameters back. After training, clients evaluate the model on their test data and report performance metrics to the server. The server aggregates these model hyperparameter updates and continuously refines the global model over multiple iterations.

### Explainable AI

The TreeSHAP algorithm is used to explain the inner workings of the Random Forest (RF) model, offering advantages over LIME, especially for tree-based models. Unlike LIME, which approximates model behavior with a surrogate model around individual predictions, TreeSHAP provides exact feature attributions based on Shapley values from cooperative game theory. These Shapley values ensure a fair and consistent distribution of feature importance by considering all possible feature combinations, resulting in more stable and reliable explanations.

TreeSHAP is specifically optimized for tree-based models, making it much more computationally efficient than LIME, especially when working with large or complex models. While LIME perturbs input data and fits a local surrogate model—introducing potential approximation errors and instability—TreeSHAP ensures exactness and consistency in its calculations. This makes it highly valuable for both local and global interpretability of tree-based models. Overall, TreeSHAP’s accuracy, efficiency, and solid foundation in Shapley values make it a better choice for producing reliable and scalable model explanations.

A Beeswarm plot visually shows Shapley values for each feature in a dataset, revealing their contributions to the model’s predictions. Each dot on the plot represents a Shapley value for a specific data point, with its position along the x-axis indicating the size and direction of the feature’s effect. The y-axis displays the evaluated feature, and features with a wider spread of dots or extreme Shapley values usually have a greater influence on the model’s predictions.

**Fig. 11:**
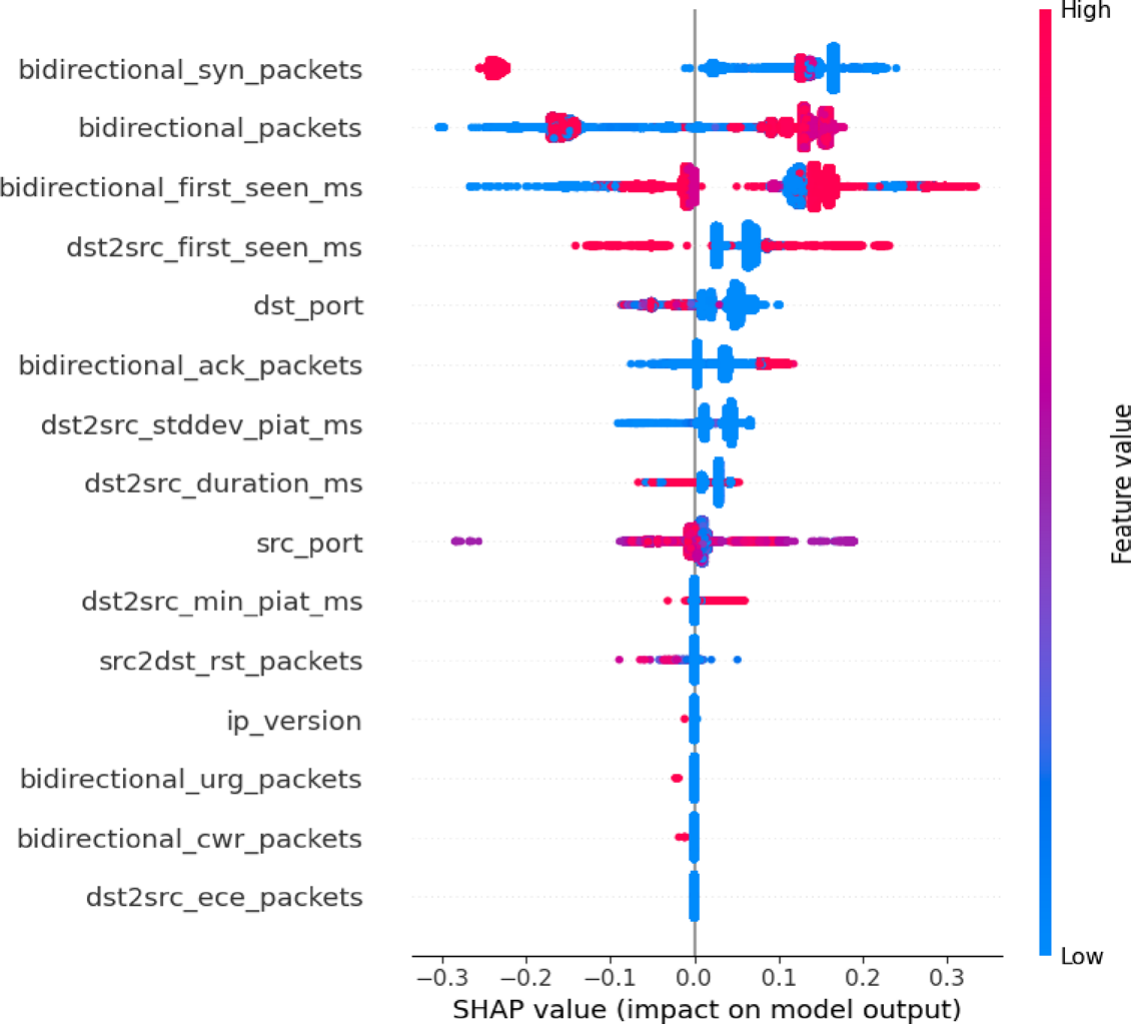
TreeSHAP algorithm scores for each feature for benign class.

**Fig. 12:**
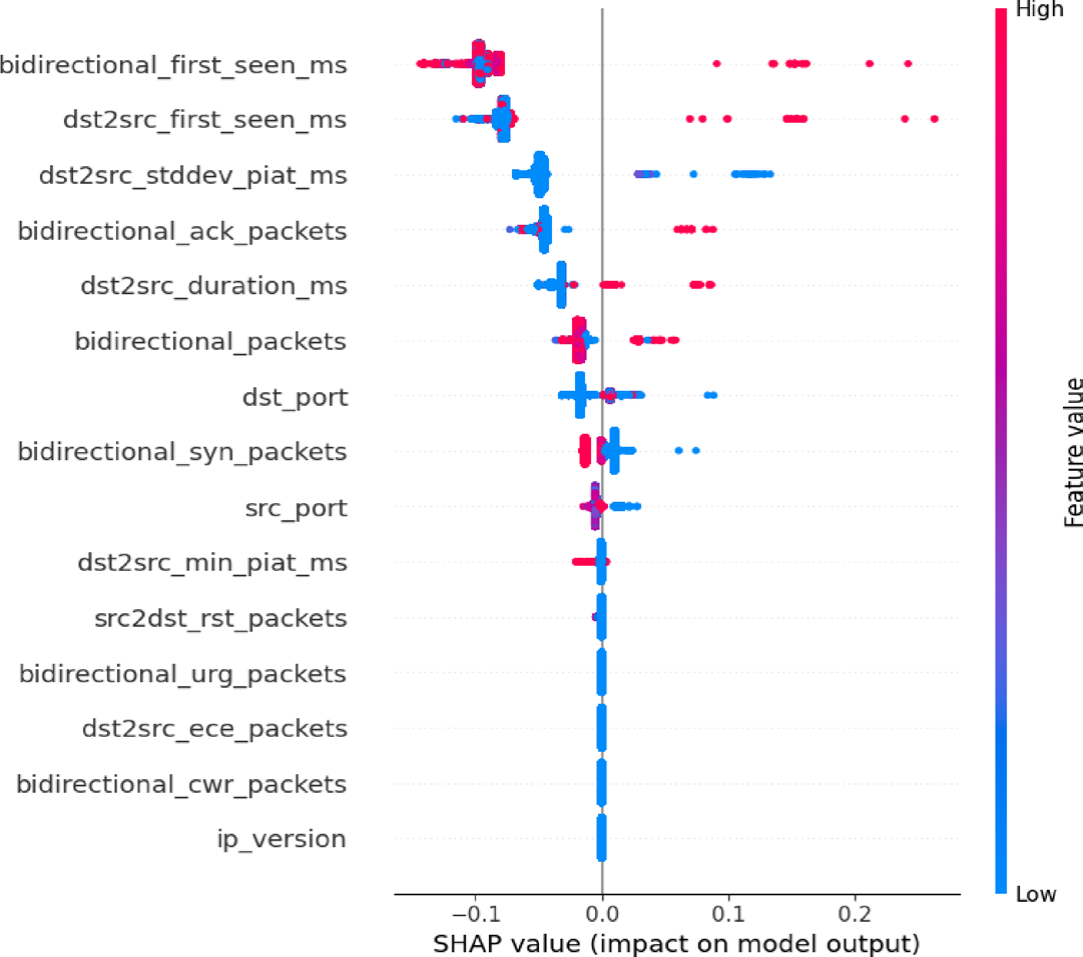
TreeSHAP algorithm scores for each feature for DoS attack class.

Figures 11 and 12 illustrates the Beeswarm plot of SHAP scores for each feature in the dataset for the benign and DoS classes. In the benign class, key features such as *bidirectional_syn_packets*,* bidirectional_packets*, and *bidirectional_first_seen_ms* exhibit the largest SHAP value ranges, indicating their dominant influence on the model’s predictions. These features serve as the primary drivers of classification decisions. Conversely, features like *bidirectional_urg_packets*,* bidirectional_cwr_packets*, and *dst2src_ece_packets* show relatively narrow SHAP distributions, suggesting minimal impact on the model’s outputs. For the DoS class, the most influential feature is *bidirectional_first_seen_ms*, signifying that the initial timing of a bidirectional flow is a crucial determinant in classification. Similarly, *dst2src_first_seen_ms* plays a significant role, reflecting flow timing from a destination-to-source perspective. Both features display wide SHAP value distributions, indicating variability in their impact across different observations. Other notable features, such as *dst2src_stddev_piat_ms* and *bidirectional_ack_packets*, contribute to a lesser extent but remain relevant, likely capturing variations in packet inter-arrival times and acknowledgment patterns that distinguish specific traffic behaviors.

#### Explainability analysis

Beyond qualitative visualizations, quantitative explainability evaluations have been integrated into FeXAI in order to assess the robustness and reliability of the produced interpretations. First, Explanation Stability (ES) was computed by adding small Gaussian noise to the input samples and calculating the Spearman correlation between the original and perturbed SHAP rankings. Stability scores ranged from 0.89 to 0.93, which states that the explanations given by the model are consistent under small variations of the input. Second, Cross-Client Consistency (CCC) was evaluated by comparing SHAP feature importance vectors across different federated clients by computing their cosine similarity. The results, with a mean consistency score of 0.91, prove that clients with heterogeneous data distributions converge to similar explanatory patterns. Finally, the Feature Importance Agreement Score (FIAS) quantifies the alignment between top features at the global federated model and aggregated local client explanations by attaining a Jaccard similarity of 0.84, confirming that federated aggregation maintains key explanatory features learned at the edge. All these metrics together support that the explanations of FeXAI are stable, consistent, and reproducible, thus reinforcing the reliability of its XAI component in real-world smart city cybersecurity applications.

### Communication overhead

To evaluate the feasibility of deploying the FeXAI framework in IoV and EVSE environments—where network bandwidth is often limited—this work quantified the communication overhead incurred during the federated learning (FL) process. For each round, clients transmit their updated model parameters to the server and subsequently receive the aggregated global model. As seen in Table 10, across two FL rounds involving three clients, the average model update size exchanged between the server and clients ranged from **96**,**439 bytes to 101**,**740 bytes**. This resulted in a total upload volume of **1**,**189**,**074 bytes** and an equal download volume of **1**,**189**,**074 bytes**, yielding a cumulative communication cost of **2**,**378**,**148 bytes (≈ 2.38 MB)** for the complete FL cycle. These results demonstrate that the communication footprint of our Random Forest–based FL architecture is extremely lightweight compared to deep learning–based FL systems, which often require tens of megabytes per round. The low per-round transfer size confirms that FeXAI can operate efficiently under constrained edge connectivity, making it practical for deployment in real-world IoV and EVSE infrastructures where devices may rely on low-bandwidth links such as 3G/4G, roadside Wi-Fi, or DSRC. The quantified overhead validates that the proposed federated learning workflow is communication-efficient and suitable for latency-sensitive and bandwidth-restricted vehicular environments.

**Table 10:** Results of federated learning communication overhead after two iterations.

Round	Avg. model update size (bytes)	Total upload (bytes)	Total download (bytes)	Total communication (bytes)
Round 1 (Training)	96,439	289,317	289,317	578,634
Round 1 (Evaluation)	96,439	289,317	289,317	578,634
Round 2 (Training)	101,740	305,220	305,220	610,440
Round 2 (Evaluation)	101,740	305,220	305,220	610,440
Total	—	**1**,**189**,**074**	**1**,**189**,**074**	**2**,**378**,**148**

The values highlighted in bold are the best-performing results for the respective evaluation metrics.

### Aggregation method selection

This work focuses on the Federated Averaging aggregation strategy because of its widespread application, simplicity, computational efficiency, and compatibility with tree-based learning models deployed to resource-constrained edge environments. FedAvg aggregates client updates using a weighted average proportional to local dataset sizes, which yields stable convergence for moderate data heterogeneity while keeping both communication and computation overhead low. These properties make FedAvg especially suitable for intrusion detection scenarios within IoV, EVSE, and VANET infrastructures where edge nodes frequently operate under limited connectivity and processing capabilities.

Given the inherently non-IID (Non-Independent and Identically Distributed) nature of the datasets considered, alternative aggregation strategies may provide enhanced robustness due to highly heterogeneous client distributions. More specifically, FedProx introduces a proximal regularization term to mitigate client drift by constraining local updates toward the global model. SCAFFOLD employs control variates for variance reduction in client updates and for speeding up convergence. FedDyn extends this notion by including dynamic regularization that aligns the client optimization objectives more closely over time. Of note, however, is that FedProx and SCAFFOLD are designed primarily for gradient-based learning paradigms; therefore, their impact is limited when applied to tree-based models like Random Forests, which do not depend on gradient optimization. Thus, in this study, these aggregation strategies are considered not for substantial performance improvement but for demonstrating compatibility with this framework.

FedAvg is selected for establishing a sound and computationally feasible baseline that aligns with the methodological focus of this work on explainability, communication efficiency, and privacy preservation. Advanced aggregation techniques will be investigated in future research combined with gradient-based models to further improve convergence stability and performance under highly heterogeneous settings.

Although FeXAI demonstrates excellent performance on different IoT security datasets, several extensions can be considered that would improve robustness and applicability in real-world smart city environments. While the current study validates each dataset separately, future research must investigate cross-dataset and cross-domain generalization, including transfer learning among IoV, EV charging systems, and VANET environments. This will facilitate assessing the resilience of the framework to domain shift and evolving attack behaviors.

### Future work

Future work should also consider federated multi-dataset training, in which each dataset acts as a different FL client to simulate large-scale and heterogeneous smart city deployments. Such experiments would confirm FeXAI’s ability to integrate knowledge among different subsystems without breaching data privacy. Again, advanced FL aggregation schemes, such as FedProx, SCAFFOLD, and FedDyn, might deal with non-IID data distribution more effectively, offering better convergence stability in heterogenous edge environments.

Another promising direction is integrating domain adaptation and representation learning to improve generalization across different communication protocols and feature spaces. Finally, while TreeSHAP provides qualitative explanations, our future work should integrate quantitative assessments of explainability, including stability of explanations, cross-client consistency, and user-centered evaluation with security analysts.

Taken together, the following set of directions can help evolve FeXAI further towards a comprehensive, adaptable, and resilient cybersecurity framework suitable for complex interlinked smart city ecosystems.

## Conclusion

This study presents FeXAI, a comprehensive and privacy-preserving cybersecurity framework designed for intrusion detection in smart city transportation and energy infrastructures. By integrating Federated Learning (FL), Random Forest (RF), and Explainable AI (TreeSHAP), the framework delivers accurate and interpretable detection of cyberattacks such as DoS, DDoS, Reconnaissance, Sybil, Replay, and Spoofing. FL enhances scalability and protects data privacy by retaining sensitive information at local edge nodes, while TreeSHAP provides transparent explanations of model behavior. Experimental evaluations on the CICIoV2024, CICEVSE2024, and VeReMi Extension datasets demonstrate consistently high detection performance, with F1 scores reaching up to 0.99. These findings highlight FeXAI’s effectiveness as a reliable and explainable cybersecurity framework for addressing threats within individual smart city subsystems.

## Data Availability

The CICIoV2024 dataset can be accessed from [https://www.unb.ca/cic/datasets/iov-dataset-2024.html](https:/www.unb.ca/cic/datasets/iov-dataset-2024.html), The CICEVSE2024 dataset can be accessed from [https://www.unb.ca/cic/datasets/evse-dataset-2024.html](https:/www.unb.ca/cic/datasets/evse-dataset-2024.html) , The VeReMi extension dataset can be accessed[https://www.sciencedirect.com/science/article/pii/S2352864822001407](https:/www.sciencedirect.com/science/article/pii/S2352864822001407).

## List of symbols

X: Original dataset or input feature space
X_missing_: Set of missing values in the dataset X
X_non−null_: Dataset with missing values removed
C_1_, C_2_, …, C_k_: Set of k distinct classes in the dataset
x: A categorical variable belonging to {C_1_, C_2_, …, C_k_}
x_encoded_: Encoded value of a categorical variable x, mapped to {0,1,2, …, k}
r_XY_: Pearson correlation coefficient between variables X and Y
X_i_, Y_i_: i^th^ data points of variables X and Y
X̄, Ȳ: Mean values of variables X and Y
n: Total number of samples
Cv: Covariance matrix of dataset X
λ: Eigenvalue of the covariance matrix Cv
v: Eigenvector corresponding to the eigenvalue λ
x_synthetic_: Synthetic data point
x_1_, x_2_: Two existing data points used for generating x_synthetic_
w_i_: Class-specific weight for class i to handle class imbalance
n_samples_: Total number of samples in the dataset
n_classes_: Total number of classes in the dataset
n_samples of class i_: Number of samples in class i
J(θ): Weighted loss function for classification
m: Number of training samples
y_ic_: Indicator variable; 1 if sample i belongs to class c, otherwise 0
$\:{h}_{\theta\:}\left({x}_{i}\right)$: Model prediction (predicted probability) for input x_i_
w_c_: Weight assigned to class c
P(C_k​_∣x): Probability that the input x belongs to class C_k_
P(C_k_): Prior probability of class C_k_
P(x_i_​∣C_k_​): Probability of the feature x_i_​ occurring under class C_k_​
d(p,q): Euclidean distance between two vectors p and q
p_i_, q_i_: i^th^ components of vectors p and q
G(t): Gini index at node t in a decision tree
P_i_: Probability of class i at a given node t
ϕ(f_i_​): Shapley value for feature f_i_​ in a feature attribution method
S: Subset of features excluding f_i_
F: Full set of features
v(S): Value function (e.g., model output) for subset S of features
Contribution (f_i_): Contribution of feature f_i_ ​ in a decision path of a tree model
p_leaf_: Probability of reaching a particular leaf node in the tree
|path|: Length of path to the leaf node from root
$\hat{y}_{{with}}$: Model prediction including feature f_i_
$\hat{y}_{{without}}$: Model prediction excluding feature f_i_
